# Causes of death among women aged 17–49 years between 2007 and 2010 in Maputo, Mozambique

**DOI:** 10.7189/jogh.07.020411

**Published:** 2017-12

**Authors:** Saara Parkkali, Bright I Nwaru, Orvalho Augusto, Fatima Abacassamo, Julie Cliff, Elina Hemminki

**Affiliations:** 1 Health and Social Care Systems, National Institute for Health and Welfare, Helsinki, Finland; 2 Krefting Research Centre, Institute of Medicine, University of Gothenburg, Gothenburg, Swede; 3 Centre for Population Health Sciences, Usher Institute of Population Health Sciences and Informatics, University of Edinburgh, Edinburgh, Scotland, United Kingdom; 4 Department of Community Health, Medical Faculty, Eduardo Mondlane University, Maputo, Mozambique

## Abstract

**Objectives:**

To describe causes of death among young women and estimate the role of HIV/AIDS as a cause in Maputo City, based on the civil death register.

**Methods:**

Death data of 17–49 year–old women were abstracted from January 2007–March 2010 from the civil death register in Maputo City, registering overall about 15 000 deaths per–year. Causes of death in the register were either based on physicians’ diagnoses on death certificates or determined by asking questions to deceased relatives. Causes of death were written in Portuguese; we translated them into English and classified them into 106 codes using ICD–9; these codes were then categorized into 10 groups. Estimated populations from the 2007 census were used to calculate annual mortality rates. An earlier study was used to compare deaths in 2001.

**Findings:**

A total of 9640 deaths (6510 for residents of Maputo City) were registered and 77% had a specified cause of death reported. HIV–deaths represented 36% of all deaths and 40% among 25–39 year–olds. The death rate did not increase linearly by age, as there was a peak among women aged 30–34 years. The overall annual death rate was 6.7 deaths per 1000 population, with a notable decline by year. Death rates for HIV slightly declined by year. HIV–deaths explained most of the peak in death rate among 30–34–year–olds. The share of HIV–deaths among all deaths increased from 18% in 2001 to 35% in 2007–2010. Sixty–eight percent of all and 92% of HIV–related deaths occurred in hospital, with no increase over time.

**Conclusions:**

Routine death register was useful to study death rates, distribution of deaths, and change over time in the urban setting of Maputo during late 2000s. Over time, the death rate among 17–49 years old women seemed to have declined, but the relative contribution of HIV increased.

Available data from various sources in Sub–Saharan Africa show that in recent decades the overall death rate has declined and the distribution of causes of deaths has changed [1,2]. Chronic diseases and violent deaths (especially traffic accidents) have partly replaced infectious diseases. However, the HIV epidemic appears to have slowed the mortality decline and increased the role of infectious diseases [1,2]. Most conclusions on death rates and causes are based on estimates derived from censuses and ad hoc studies, as death registration is incomplete or content unreliable [1–3]. With the exception of South Africa, there are few studies on causes of death using civil death registration systems [4–7].

Maputo City in Mozambique has a death registration system, but the data have been used sparingly for health statistics or research. There is one study [8] from 1994 and another published as an internal report in 2001 [9]. The first purpose of this study was to describe the recorded causes of death among 17–49 year–old women in Maputo City in 2007–2010 using the civil death registration system. Second, we aimed to estimate the role of HIV/AIDS as a cause of death. Furthermore, we estimated overall death rates, calculated the proportion of deaths occurring in hospital, and compared the causes of deaths to those reported in a 2001 study [9]. The data are a spin–off from a trial [10], for which the outcomes of the study women were searched from various sources.

## CONTEXT

Mozambique is a low income country in Sub–Saharan Africa. After the end of the long civil war in 1992, Mozambique’s economic development gradually recovered, and the health of the population improved, with the exception of a severe HIV epidemic. In 2010 national estimates of HIV–prevalence among 15–49 year–olds were 13.8% and among pregnant women 15.8% [11]. However, UNAIDS–adjusted estimates for adults, taking into account the population structure, were somewhat lower (11% in 2011) [12].

Maputo City is the capital of Mozambique, located in the southern region with more than one million residents. In Maputo, societal and health indicators are notably better than in the rest of Mozambique. In 2012, the GDP per capita was almost double that of the average national level (US$ 1 153 vs 593) and illiteracy rate was only 6% vs 33% nationally [13]. Due to the civil war, Maputo was isolated from the rest of the country until 1992; as a consequence HIV arrived there late [8], but it spread rapidly. It has been estimated that in the whole southern region (including Maputo) the prevalence of HIV in 2010 among 15–49 year–old women was 21% [14]. In Maputo, in a cohort of pregnant women in 2006–2008 recruited into a pragmatic randomized controlled trial on iron prophylaxis in Maputo, the HIV–positivity was 20% [10].

At the time of the iron study, pregnant women in Maputo were eligible for and utilized prenatal care (unpublished data from local experts), and HIV testing was routinely offered the whole country [15]. In Mozambique, the guideline on the organization of HIV–treatment was revised during the years of the study [12,16]. To our knowledge, HIV–positive women were given antiretroviral drugs in some Maputo prenatal centers, but not in other prenatal centers (unpublished data from local experts). It has been estimated that in the southern region the coverage of prevention of mother to child transmission of HIV was 73% in 2010 [14]. Outside pregnancy, HIV–testing was self–initiated and antiretroviral drug treatment was available through designated clinics.

## METHODS

Originally the death data were collected to complete the tracing of 4326 pregnant women recruited for a trial in Maputo [10]. In that trial, the effects of two iron administration policies (routine iron prophylaxis vs screening and treatment for anemia during pregnancy) on maternal and child health were compared. The results from the trial have been presented in a previous paper [10].

Ethical approval for the study was obtained from the Mozambique Ministry of Health Ethics Committee (CNBS [Ref. 84/CNBS/06]) and Eduardo Mondlane University Medical Faculty Ethics Board (Jan 25, 2006) [10]. A positive statement was obtained from the National Research and Development Centre for Welfare and Health (STAKES), Helsinki, Finland (Dno 2571/501/2007). Oral and written informed consent was obtained from participating women [10].

The deaths of all 17–49 year–old women registered in the Maputo City civil death register (Registo Civil) from January 2007 to March 2010 were included in the study. The death registration system in Maputo is described in **Online Supplementary Document(Online Supplementary Document).** In brief, there was only one civil death register (Registo Civil) in Maputo City, registering overall about 15 000 deaths per year. Deaths were registered by the location of death, and many deaths of people from nearby areas were registered in Maputo City. If the death occurred at home, relatives reported it to a civil administrative office, where a letter of declaration of death was written. The relatives took the letter to the Registo Civil office, where a death report was written and a death certificate issued. If the death occurred in a health facility, the death certificate was filled in by a physician. The death certificate was taken to the Registo Civil by the relatives or a person from the Registo Civil located in a mortuary or in hospital. In the Registo Civil death reports were first piled as loose papers and later compiled into books. All documents were handwritten (**Online Supplementary Document(Online Supplementary Document)**).

The cause of death was reported in death reports (**Online Supplementary Document(Online Supplementary Document)**). In the case of a hospital death a physician wrote the cause on the death certificate, and the Registo Civil official copied it into the death report. If the deceased relatives had no death certificate, the Registo Civil official asked certain questions to elicit the causes of death.

For this study we collected the following information from the death reports in Registo Civil: register number, age, date of birth, residence, date of death, place of death (hospital/out of hospital), date of death registration, basic cause of death (written in Portuguese or a local abbreviation code). Data were then entered by hand on data collection sheets, and later computerized into Microsoft Access 2000 database. We did not very the causes of death, but took the information as noted in the death register.

### Classification of causes of deaths

Causes of deaths were hand written in Portuguese, either as diagnoses, lay terms or by local abbreviation codes. The different terms (about 1000 and 534 when similar terms were combined) were translated into English (a Finnish researcher with nursing background, fluent in both Portuguese and English, SP).

The English terms and the local abbreviation codes were classified using the 9th version of the International Classification of Diseases and Related Health Problems (ICD–9 codes) into 106 codes. The coding was made by a physician (EH), discussing with other researchers. The most common abbreviations were clarified by the local researchers. The ICD codes were grouped into 18 and 10 groups (see Results) to reflect the likely cause of death, taking into account the inaccuracy at the registration stage. Mostly only one cause of death was given. In a few cases two causes were given, eg, HIV and tuberculosis. In that particular case, we classified the cause as HIV. All classifications were made solely on the basis of the causes of death and women’s background characteristics were not used in the coding process.

### Statistical analysis

For analysis, the data in Microsoft Access 2000 format was transformed into SPSS. Cross–tabulations of the grouped causes of deaths were made by age and year, and the proportions of HIV deaths were calculated. In the 2007 death register books, 12 women died in 2006: they were included in the total deaths, but excluded from yearly data. Maputo City residents were defined by the information in the Register.

To calculate annual mortality rates for Maputo City residents, the projected population numbers from the Instituto Nacional de Estatistica (INE) were used [17]. The INE used 2007 census numbers to make projections for the coming 40 years. The projected numbers were given by 5–year age groups, sex and province, separating Maputo City, as Maputo City is informally treated as an independent province. We calculated the total death rates (per 1000 population) across age groups by dividing the total number of deaths by the population size of each age group. Similarly, HIV–related death rates (per 1000 population) were calculated across age groups by dividing the number of HIV–related deaths by the population size of each corresponding age group. We calculated the confidence interval for the death rates using the Wilson score method [18].

At the time of the data collection, death data for 2010 was available for the first three months of the year. For calculating the annual HIV–related death rates for 2010, we multiplied the death rate by four. As some deaths, particularly malaria, are sensitive to the time of year, the 2010 results are given with reservation (see Results).

To compare our results to those reported in 2001 we relied on a table describing deaths of 15–44 year–old women, who had been registered in the Maputo City death register [9]. To make our study population more similar to the 2001 population, women aged 45–49 were excluded. The grouping of causes of deaths was modified so that it matches the grouping used in the 2001 study, which was based on the Burden of Disease Study categories [9].

## RESULTS

Altogether 9643 deaths among 17–49 year–old women were recorded in the Maputo City death register, of whom 6513 were Maputo residents. Three women aged less than 17 years were excluded, leaving 6510 women.

The causes of death categorized into 10 groups are given by age in **Table 1** and in more detail in Table S1 in **Online Supplementary Document(Online Supplementary Document)**. Most deaths had a cause registered and only in 0.5% of deaths was the cause lacking. But in 22% of deaths the cause was ill–defined or we could not translate it into a disease class. HIV–related deaths represented over a third of deaths, being close to 40% among 25–39 year–old women. Tuberculosis was recorded as the cause of death in 8%, malaria in 5% and other infections in 8% of women. Chronic diseases represented 12% of deaths, but their share increased notably by age. Violent (non–disease) deaths represented about 2% of deaths (5% among the youngest age group, 17–24 year–olds), and more than half of them were due to intentional violence.

**Table 1:** Distribution of deaths (%) by age and cause of death, 17–49 year–old women, Maputo residents, 2007 – March 2010

Death cause	Age group						
**17–24**	**25–29**	**30–34**	**35–39**	**40–44**	**45–49**	**Total**
Total number of deaths	1061	1421	1371	1043	856	758	6510
HIV	317 (29.9)	551 (38.8)	556 (40.5)	407 (39.0)	272 (31.8)	219 (28.9)	2322 (35.7)
Tuberculosis	93 (8.8)	121 (8.5)	127 (9.3)	89 (8.5)	69 (8.1)	50 (6.6)	549 (8.4)
Malaria	72 (6.8)	69 (4.9)	77 (5.6)	41 (3.9)	46 (5.4)	38 (5.0)	343 (5.3)
Other infections	117 (11.0)	118 (8.3)	105 (7.7)	61 (5.8)	55 (6.4)	49 (6.5)	505 (7.8)
Anemia, malnutrition	36 (3.4)	57 (4.0)	43 (3.1)	30 (2.9)	28 (3.3)	27 (3.6)	221 (3.4)
Pregnancy related	35 (3.3)	42 (3.0)	26 (1.9)	20 (1.9)	18 (2.1)	6 (0.8)	147 (2.3)
Chronic diseases	115 (10.8)	113 (7.9)	126 (9.2)	129 (12.4)	142 (16.6)	164 (21.6)	789 (12.1)
Violent death	51 (4.8)	25 (1.8)	23 (1.7)	22 (2.1)	15 (1.7)	12 (1.6)	148 (2.3)
Not clear*	221 (20.8)	316 (22.2)	43 (3.1)	280 (20.4)	209 (24.4)	188 (24.8)	1453 (22.3)
No information†	4 (0.4)	9 (0.6)	26 (1.9)	8 (0.6)	2 (0.2)	5 (0.7)	33 (0.5)
Total %	100.0	100.0	100.0	100.0	100.0	100.0	100.0

*Ill–defined causes, unknown abbreviation.

†No cause of death.

**Table 2** gives the estimated death rates by age. The overall annual death rate was 6.7 deaths per 1000 population. The death rate did not increase linearly by age, as there was a peak among 30–34–year–old women. Only the oldest age group (45–49 year–old) had a higher death rate than the 30–34 year–old women. HIV–related deaths explained more than a third of the peak among the 30–34 year–old women.

**Table 2:** Estimated annual death rates by age, 17–49 year–old women, Maputo residents, 2007 – March 2010

	Age group						
**17–24**	**25–29**	**30–34**	**35–39**	**40–44**	**45–49**	**Total**
Population in 2007–2010*	338 864	183 206	150 841	124 659	96 065	74 907	968 541
Number of deaths	1061	1421	1371	1043	856	758	6 510
Death rate per 1000 per year (95% CI)	3.13 (2.95–3.32)†	7.76 (7.36–8.17)	9.09 (8.62–9.58)	8.37 (7.88–8.89)	8.91 (8.34–9.52)	10.12 (9.43–10.86)	6.72 (6.56–6.89)
Number of HIV deaths	317	551	556	407	272	219	2 322
HIV death rate per 1000 per year (95% CI)	0.94 (0.84–1.04)†	3.01 (2.77–3.27)	3.69 (3.39–4.00)	3.26 (2.96–3.60)	2.83 (2.52–3.20)	2.92	2.39 (2.30–2.50)

CI – confidence interval

*Projected populations (women): sum of 2007, 2008, 2009 and a quarter of 2010 projected populations.

†15–24 year–old population × 8/10.

**Table 3** gives the estimated death rates by year. The overall death rate notably declined by year, from 7.2 per 1000 population in 2007 to 6.4 in 2009. The rate in 2010 covers only the first three months. HIV death rates also declined during the period (**Table 3**).

**Table 3:** Rates and proportions of deaths due to HIV, by year, 17–49 year–old women, Maputo residents*

	2007	2008	2009	2010†
Population	290 975	297 012	303 189	309 460
Total deaths, n	2081	2091	1938	1584
death rate per 1000 (95% CI)	7.15 (6.85–7.46)	7.04 (6.75–7.35)	6.39 (6.11–6.68)	5.12 (4.90–5.40)
HIV deaths, n	883	675	620	572
HIV deaths, % of deaths (95% CI)	42.4 (40.3–44.6)	32.3 (30.3–34.3)	32.0 (29.9–34.1)	36.1 (33.8–38.5)
HIV death rate per 1000 (95% CI)	3.03 (2.84–3.24)	2.27 (2.11–2.45)	2.04 (1.89–2.21)	1.85 (1.70–2.01)

CI – confidence interval

*23 deaths excluded from the analysis as their date of death was missing.

^†^In 2010 the numbers of deaths in the first three months were multiplied by 4.The numbers which may be sensitive to time of year are given in square parenthesis.

We also inspected the time–trends by age and year (Table S2 in **Online Supplementary Document(Online Supplementary Document)**). In the two youngest age groups death rates declined over time, but in the older age groups the trends were not systematic. HIV–death rates declined in the three youngest age groups and among the 40–44–year–olds, but among the 35–39 and 45–49 year–olds there was a U–shaped trend.

**Table 4** gives the distribution of deaths by the place of death, specifying HIV–deaths. In all years, most deaths (overall 92%) diagnosed as HIV–related, had occurred in a hospital. The proportion of other deaths occurring in hospital was only a little over half, with no increase over time. In each year the difference between HIV–deaths and other deaths is statistically significant (*P*<0.001).

**Table 4:** Proportions (%) of HIV and other deaths by death place and year, 17–49 year–old women, Maputo residents*

	2007	2008	2009	2010†	Total
**No. HIV deaths:**	883	675	620	143	2 321
In hospital	92.0	92.9	91.0	93.7	92.1
Elsewhere	8.0	7.1	9.0	6.3	7.9
Total %	100.0	100.0	100.0	100.0	100.0
**Other deaths:**	1198	1416	1318	253	4185
In hospital	53.7	54.7	54.6	52.2	54.2
Elsewhere	46.3	45.3	45.4	47.8	45.8
Total %	100.0	100.0	100.0	100.0	100.0
**No. total deaths:**	2081	2091	1938	396	6506
In hospital	69.9	67.0	66.2	67.2	67.7
Elsewhere	30.1	33.0	33.7	32.8	32.3
Total %	100.0	100.0	100.0	100.0	100.0

*23 deaths excluded from the analysis as their date of death was missing

†First three months. In each year the difference between HIV–deaths and other deaths in regard to the place of death is statistically significant (*P*<0.001)

**Table 5** compares the proportions of selected causes of deaths in Maputo death register in 2001 and 2007–March 2010. The 2001 data concerns 15–44–year–olds and were obtained from a published report [9]. In 2007–2010, data for Maputo residents only are also shown. The selected causes of deaths covered a much higher proportion in 2001 (81%) than in 2007–10 (63%). The share of HIV as a cause of death had notably increased during this short time period (from 18% to 35%), and that of tuberculosis and particularly malaria declined. The information in 2007–10 was relatively similar whether the analysis included all registered deaths or only Maputo residents.

**Table 5:** Comparison of selected causes of deaths among 15–44 year–old women in 2001 in Maputo death register to those among 17–44 year–old women in Maputo death register in 2007 – March 2010 and to Maputo residents in 2007 – March 2010*

	2001 Death Register		2007–2010 Death Register**†**		2007–2010 residents**†**	
**Causes of death**	Number	%	Number	%	Number	%
HIV	370	17.7	3367	34.9	2322	35.6
Tuberculosis	355	17.0	820	8.5	550	8.4
Malaria	474	22.7	476	4.9	344	5.3
Diarrhea	88	4.2	196	2.0	113	1.7
Pneumonia	72	3.4	147	1.5	105	1.6
Meningitis/Brain infection	36	1.7	173	1.8	120	1.8
Anemia	120	5.7	319	3.3	221	3.4
Maternal causes/Pregnancy related	70	3.4	220	2.3	147	2.3
Hypertension	57	2.7	278	2.9	184	2.8
Traffic accidents	51	2.4	44	0.5	21	0.3
Total		81.0		62.7		63.4
Total deaths	2090	100.0	9637	100.0	6510	100.0

*Only causes with specific diagnosis in the denominator (excluding deaths with missing or ill–defined cause).

†First three months in 2010.

## DISCUSSION

Our study in an urban setting, Maputo in Mozambique, showed that overall the death rate among 17–49–year–old women declined from 2007–2010, the highest death rate was among 30–34–year–olds, and HIV was recorded as the cause of death in over a third of deaths. HIV–deaths explained most of the decline over time and observed variation by age. Other infections were common causes among younger women, but among women older than 40 years, chronic diseases as causes of death became more prevalent. Comparison to an older study suggests that the role of HIV as the cause of death had increased from 2001.

### Can the results be trusted?

There is no good estimate of the coverage of registering deaths in Maputo. In a study from 1994, the coverage was estimated to be 86% [8]. The coverage at the end of 2000s may be higher. A death certificate is needed to get a burial place in a cemetery. In the city, burial space outside cemeteries is scare. Furthermore, relatives need the death certificate to receive money for pension and other benefits.

The recorded cause of death could be obtained technically by studying the register, but did the recorded death represent the real cause? There is no published validity study available. Two thirds of deaths, and over 90% of those recorded as HIV–deaths, had occurred in hospital. In hospital deaths, health personnel, mainly physicians, were involved. However, for one third of deaths, civil servants without medical background, even though trained to elicit replies to questions, determined the cause from relatives’ descriptions. The method of verbal autopsy, if well–performed, can be useful for determining causes of death [18,19]. Furthermore, the results look plausible: the death rate increased and chronic diseases became more prevalent by age.

In South–Africa it has been suggested that to avoid social problems physicians do not write HIV for the cause of death [5]. In the Maputo death register, a HIV–diagnosis was common and in addition various abbreviations, which were not transparent for the lay–person, such as ODV (outras doenças por virus, other viral diseases) were used to indicate HIV. Studies from South–Africa show both poor coverage and low accuracy of causes of death in the civil death registration system [5–7]. However, the death registration system in Maputo is very different to that in South Africa, and comparison is not very helpful.

A limitation of our study is that we had an incomplete data for 2010, therefore given seasonal variations across the year, our conclusion may not be applicable to the whole year. The 2001 study was used to compare our findings to an earlier time. Unlike our study the 2001 study included women 15–17 years of age. This small difference in the compared populations is unlikely to explain the large differences in causes of death between the two time periods.

### Comments on result details

HIV–infection was classified as the cause in over a third of deaths. This may be an underestimate, as HIV could have been behind a number of other deaths. If other causes were reported in addition to HIV, we classified the death as HIV. It was, however, rare to have multiple diagnoses. In a 2007 study based on census interviews, causes of death in the previous year were enquired about using a verbal autopsy method [11]. In that study almost half of adults dying of HIV had also had tuberculosis, and most persons who had tuberculosis as the cause of death also had HIV.

The relative contribution of HIV–infection had increased from 2001. But as the source we used for 2001 did not give comparable population numbers, we do not know if rates of HIV deaths had increased and if so, to what extent. In the time–period 2007–2010 there was a declining death rate overall and specifically for HIV. Death rates did not vary linearly by age and the highest rate was among 30–34–year–old women. The variation by age may be due to varying infection prevalence in different age cohorts or varying rates of HIV treatment. The future trend of HIV deaths among Maputo women is difficult to predict. In a cohort of pregnant women in 2006–2008, the HIV–positivity was 20%, and the highest rate was among 25–29–year–olds (28%) [10].

About 3% of causes of death were classified as anemia and/or malnutrition; some of these deaths may have been due to malaria, HIV or other infections. In our study, 1.7% of deaths were classified to be due to diarrhea. This is much less than in the 2001 study (4.2%) [9] or in a 1994 study (8.8% among 15–59 old women) [8]. Among older women, chronic diseases were common. Violent deaths represented 2.3% of causes of death, which agrees with previous findings showing that violent deaths are much less common among women than men [2]. A high share of violent causes of deaths were intentional violence (interpersonal violence and suicides). Furthermore, it is likely to be an underestimate as unclear cases were coded as non–intentional.

In addition to the current study and the one in 2001 [9] we found one older study from 1994 using death register data [8]. In the 1994 study, among 15–59–year–old women the ten leading deaths included malaria (11%) tuberculosis (9.8%), violent deaths (8.8%), diarrheal diseases (8.8%), but not HIV; the authors discuss that HIV has been underreported. However, the prevalence study in prenatal clinics showed only 2.7% of pregnant women to be HIV–positive, much less than in 2008–2010. Therefore, it is very likely that the role of HIV as a cause of death has notably increased and that of other infections has decreased since 1994. The high proportion of violent deaths in 1994 may be related to the close proximity of the long civil war, which ended in 1992. In a previous study using verbal autopsy to identify maternal deaths in a small series of subjects, HIV accounted for a higher proportion (52%) of maternal deaths than our study [20].

### Further research

There are lack of data on trends in mortality levels and causes of death in Sub–Saharan Africa. Data from civil registration could serve as a useful data source to capture time trends and differences between the population groups. Using a similar approach to that employed in this study in other places may provide better understanding of the trends of death and their causes across Sub–Saharan Africa.

### Implications for practice

Computerizing data on civil registration of deaths would allow up–to–date surveillance and future projections. The basic data to be collected should be simple and easy for lay people to record. A written diagnosis should be retained and coding done afterwards. To calculate death rates, population numbers by residence should be readily available. Studies using death registration may stimulate improving death registration.

## CONCLUSION

HIV–deaths represented 36% of all deaths. The overall death rate did not increase linearly by age, as there was a peak among women aged 30–34 years, explained by HIV–deaths. Over time the death rate among 17–49 years old women seemed to have declined, but the relative contribution of HIV increased. The routine death register was useful to study the death rates, distribution of causes of deaths, and change over time in the urban setting of Maputo during the late 2000s.
