# Supplementary material for: Causes of death among women aged 17–49 years between 2007 and 2010 in Maputo, Mozambique
Source: J Glob Health. 2017 Dec 3;7(2):020411. doi: 10.7189/jogh.07.020411 (PMC5735779; doi:10.7189/jogh.07.020411)
Supplement: Online Supplementary Document [file jogh-07-020411-s001.pdf]

Online Supplementary Document

Parkalli et al. Causes of death among women aged 17–49 years between 2007 and 2010 in Maputo, Mozambique

J Glob Health 2017;7:020411

Death registration in Maputo

Mozambique is a former Portuguese colony, and unlike many other African countries it has a long tradition of civil registration.[1] Vital events, such as births, deaths, and marriages, have been registered into Registo Civil.[1] The main purpose of the vital registration has been to obtain identity cards and in case of deaths, documents needed for funerals, pension claims and settling an inheritance.[1] The civil war destroyed much of the registration system, but in urban areas outside the battles, such as Maputo, the civil registration continued to function.

In Maputo, at the time of the study, bodies of dead people, regardless of the place of death were usually taken to a morgue (casa mortuaria), where the body was kept until the funeral. The morgue was a government facility, which charged a small sum for its services. All paper work relating to the funerals was done there, and funeral agencies could be involved. If wished, or for legal reasons, the body was sent to the Pathology or MedicoLegal Department to do an autopsy. Otherwise, the body was transferred from the morgue to a cemetery.

If the death occurred at home, relatives first reported it to the nearest administrative office, where a letter of declaration of death was written. The relatives took the letter to the Registo Civil (Conservatoria do Registo Civil, civil death registration office), where a death report was written and a death certificate (boletim de óbito) was issued to the relatives. In bigger morgues there could be a person from Registo Civil writing and issuing death certificates. Since 2004, there has been a requirement that even in case of deaths at home, health authorities should be involved in determining the cause of death (Ministério da Justiça 2014). However, this seems not to happen in practice in Maputo.

If the death occurred in a health facility, the death certificate was filled in by a health worker (usually a physician). The process of the information on death reaching the Registo Civil for registration varied: the relatives took the death certificate there; in bigger morgues a person from Registo Civil could be present and issue the death report; in bigger hospitals a person from Registo Civil could be present to issue the death report.

All documents were handwritten. In the Registo Civil death reports were first piled as loose papers, each pile consisting of 300 reports, organized by date. Piles were ordered by the place where the death report had been made. These reports were then compiled into books (assento de obito), each book containing 300 deaths. Each year the numbering per place started from number one.

In case of hospital deaths, physicians wrote the cause of death on the death certificate based on clinical data. In 2009, the format of the death certificate was changed and ICD-10 adopted. Officials in the Registo Civil copied physicians' causes of death into death reports. If there was

no death certificate (e.g. death occurred at home), the officials of Registo Civil had been trained to ask questions to elicit the cause of death.

There was only one civil death register (Registo Civil) in Maputo City, registering overall about 15,000 deaths per year. As deaths were registered by the location of death, many deaths of people from the nearby areas were registered in Maputo City, as they had died in Maputo City hospitals. A death certificate was needed to obtain a burial place in a cemetery. In the city burial space outside cemeteries was scare. Furthermore, relatives needed the death certificate to access pensions and other benefits.

Supplementary Tables

Table S1. Distribution of deaths (%) by causes of death and age at death 2007- March 2010[4], detailed grouping, Maputo residents.

	17-24	25-29	30-34	35-39	40-44	45-49	total
(number of deaths)	(1061)	(1421)	(1371)	(1043)	(856)	(758)	(6510)
Death cause							
HIV	317 (29.9)	551 (38.8)	556 (40.5)	407 (39.0)	272 (31.8)	219 (28.9)	2322 (35.7)
Tuberculosis	93 (8.8)	121 (8.5)	127 (9.3)	89 (8.5)	69 (8.1)	50 (6.6)	549 (8.4)
Malaria	72 (6.8)	69 (4.9)	77 (5.6)	41 (3.9)	46 (5.4)	38 (5.0)	343 (5.3)
Diarrhea	28 (2.6)	23 (1.6)	25 (1.8)	16 (1.5)	8 (0.9)	13 (1.7)	113 (1.7)
Respiratory infection	30 (2.8)	34 (2.4)	24 (1.7)	10 (1.0)	19 (2.2)	9 (1.2)	126 (1.9)
Brain infection	24 (2.3)	32 (2.2)	32 (2.3)	8 (0.8)	13 (1.5)	11 (1.4)	120 (1.8)
Other infection	35 (3.3)	29 (2.0)	24 (1.7)	27 (2.6)	15 (1.7)	16 (2.1)	146 (2.2)
Anemia. malnutrition	36 (3.4)	57 (4.0)	43 (3.1)	30 (2.9)	28 (3.3)	27 (3.6)	221 (3.4)
Pregnancy related	35 (3.3)	42 (3.0)	26 (1.9)	20 (1.9)	18 (2.1)	6 (0.8)	147 (2.3)
Diabetes	7 (0.7)	2 (0.1)	6 (0.4)	10 (1.0)	5 (0.6)	9 (1.2)	39 (0.6)
Cardiovascular	30 (2.8)	39 (2.7)	34 (2.5)	43 (4.1)	52 (6.19	64 (8.4)	262 (4.0)
Pulmonary	33 (3.1)	39 (2.7)	34 (2.5)	30 (2.9)	17 (2.0)	19 (2.5)	172 (2.6)
Cancer	13 (1.2)	11 (0.8)	24 (1.7)	30 (2.9)	42 (4.9)	47 (6.2)	167 (2.6)
Other (chronic) diseases	32 (3.0)	22 (1.5)	28 (2.0)	16 (1.5)	26 (3.0)	25 (3.3)	149 (2.3)
Violent, intentional[3]	29 (2.7)	10 (0.7)	12 (0.9)	11 (1.0)	6 (0.7)	9 (1.2)	77 (1.2)
Violent, other [3]	22 (2.1)	15 (1.1)	11 (0.8)	11 (1.0)	9 (1.0)	3 (0.4)	71 (1.1)
Not clear[1]	221 (20.8)	316 (22.2)	280 (20.4)	239 (22.9)	209 (24.4)	188 (24.8)	1453 (22.3)
No information[2]	4 (0.4)	9 (0.6)	8 (0.6)	5 (0.5)	2 (0.2)	5 (0.7)	33 (0.5)
Total	100	100	100	100	100	100	100

[1] Ill-defined causes, unknown abbreviation.
[2] No cause of death.
[3] Intentional= interpersonal violence and suicide, other= non-intentional and not clear.
[4] Three deaths excluded from the analysis as their age at death was uncertain.

Table S2 Numbers and proportions of deaths due to HIV, by year and age, 17-49 year old women, Maputo residents[1]

	Age groups						
	17-24[3]	25-29	30-34	35-39	40-44	45-49	Total[2]
2007							
Population	102 134	55 169	45 585	37 429	28 245	22 413	290 975
total deaths, n	355	476	405	326	290	229	2 081
death rate per 1000 (95% CI)	3.48 (3.13-3.86)	8.63 (7.89-9.43)	8.88 (8.06-9.79)	8.71 (7.82-9.70)	10.27 (9.16-11.51)	10.22 (8.98-11.62)	7.15 (6.85-7.46)
HIV deaths, n	116	215	200	153	115	84	883
HIV deaths. % of deaths (95% CI)	32.7 (28.0-37.7)	45.2 (40.7-49.7)	49.4 (44.5-54.2)	46.9 (41.6-52.4)	39.7 (34.2-45.4)	36.7 (30.7-43.1)	42.4 (40.3-44.6)
HIV death rate per 1000 (95% CI)	1.14 (0.95-1.36)	3.90 (3.41-4.45)	4.39 (3.82-5.04)	4.09 (3.49-4.79)	4.07 (3.39-4.88)	3.75 (3.03-4.64)	3.03 (2.84-3.24)
2008							
Population	104 043	56 135	46 209	38 320	29 314	22 991	297 012
total deaths, n	362	461	466	328	249	229	2 095
death rate per 1000 (95% CI)	3.48 (3.14-3.86)	8.21 (7.50-8.99)	10.08 (9.21-11.04)	8.56 (7.69-9.53)	8.49 (7.51-9.61)	9.96 (8.76-11.33)	7.04 (6.76-7.36)
HIV deaths. n	103	164	178	102	69	59	675
HIV deaths, % of deaths (95% CI)	28.5 (24.0-33.3)	35.6 (31.3-40.0)	38.2 (33.9-42.7)	31.1 (26.3-36.3)	27.7 (22.5-33.6)	25.8 (20.5-31.8)	32.3 (30.2-34.2)
HIV death rate per 1000 (95% CI)	0.99 (0.82-1.20)	2.92 (2.51-3.40)	3.85 (3.33-4.46)	2.66 (2.19-3.23)	2.35 (1.86-2.98)	2.57 (1.99-3.31)	2.27 (2.11-2.45)
2009							
Population	105 822	57 270	47 041	39 014	30 544	23 498	303 189
total deaths, n	288	409	419	320	265	237	1 938
death rate per 1000 (95% CI)	2.72 (2.24-3.05)	7.14 (6.48-7.87)	8.91 (8.10-9.80)	8.20 (7.35-9.15)	8.68 (7.70-9.78)	10.09 (8.89-11.45)	6.39 (6.11-6.68)
HIV deaths, n	82	143	143	119	73	60	620
HIV deaths, % of deaths (95% CI)	28.5 (23.6-33.9)	35.0 (30.5-39.7)	34.1 (29.7-38.8)	37.2 (32.1-42.6)	27.5 (22.5-33.2)	25.3 (20.2-31.2)	32.0 (29.9-34.1)
HIV death rate per 1000 (95% CI)	0.77 (0.62-0.96)	2.45 (2.12-2.94)	3.04 (2.58-3.58)	3.05 (2.55-3.65)	2.39 (1.90-3.00)	2.55 (1.98-3.29)	2.04 (1.89-2.21)
2010[2]							
Population)	107 459	58 526	48 023	39 585	31 849	24 018	309 460
total deaths, n	(224)	(300)	(324)	(276)	(208)	(252)	(1 584)
death rate per 1000 (95% CI)	(2.08 [1.80-2.40])	(5.13 [4.60-5.70])	(6.75 [6.10-7.50])	(6.97 [6.20-7.80])	(6.53 [5.70-7.50])	(10.49 [9.3-11.9])	(5.12 [4.90-5.40])
HIV deaths, n	72	124	123	120	68	68	572
HIV deaths, % of deaths (95% CI)	(32.1 [26.4-38.5])	(41.3 [35.9-47.0])	(38.3 [32.9-43.4])	(43.5 [37.8-49.4])	(32.7 [26.7-39.3])	(27.0 [21.9-32.8])	(36.1 [33.8-38.5])
HIV death rate per 1000 (95% CI)	0.67 (0.53-0.84)	2.12 (1.78-2.53)	2.56 (2.15-3.05)	3.03 (2.54-3.62)	2.14 (1.68-2.71)	2.83 (2.23-3.59)	1.85 (1.70-2.01)

[1] 23 deaths excluded from the analysis as their date of death was uncertain.
[2] in 2010 the numbers sensitive to time of year are given in square parenthesis ; HIV deaths in the first three months are multiplied by 4.

[3] 0.8 x (population aged 15-19 years) + population 20-24 years.

Supplementary References

1. Brunborg H, Aurbakken E. Evaluation of systems for registration and identification of persons in Mozambique. Statistics Norway, Research Development. May 1997.
